# H–d-Phe–d-Pro–Gly methyl ester hydro­chloride monohydrate

**DOI:** 10.1107/S160053680800528X

**Published:** 2008-03-12

**Authors:** Mitsunobu Doi, Yuko Ichimiya, Akiko Asano

**Affiliations:** a Osaka University of Pharmaceutical Sciences, 4-20-1 Nasahara, Takatsuki, Osaka 569-1094, Japan

## Abstract

The conformation of the title tripeptide methyl ester hydro­chloride monohydrate, 1-[2-(methoxycarbonylmethylaminocarbonyl)pyrrolidin-1-ylcarbonyl]-2-phenylethanaminium chloride monohydrate, C_17_H_24_N_3_O_4_^+^·Cl^−^·H_2_O, is extended, but the structure cannot be classified as any typical secondary structure. Interactions through water molecules and chloride ions were formed, in addition to peptide–peptide hydrogen bonds, stabilizing the molecular packing. In comparison with the previous β-turn structure of the Phe–d-Pro–Gly analogue [Doi, Ichimiya & Asano (2007 ▶). *Acta Cryst*. E**63**, o4691], it was suggested that the difference between the chiralities of Phe and Pro residues of the title compound is important to induce the β-turn structure.

## Related literature

For related literature, see: Cremer & Pople (1975 ▶); Doi, Fujita *et al.* (2001 ▶); Doi, Ichimiya *et al.* (2007 ▶); Espinosa & Gellman (2000 ▶); Llamas-Saiz *et al.* (2007 ▶); Tamaki *et al.* (1985 ▶); Yamada *et al.* (2002 ▶).
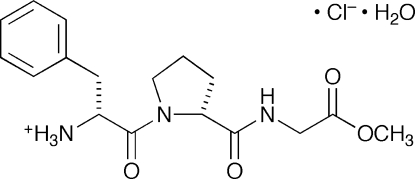


## Experimental

### 

#### Crystal data

C_17_H_24_N_3_O_4_^+^·Cl^−^·H_2_O
*M* *_r_* = 387.86
Orthorhombic, 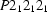

*a* = 7.3707 (5) Å
*b* = 9.6667 (7) Å
*c* = 27.099 (2) Å
*V* = 1930.8 (2) Å^3^
*Z* = 4
Mo *K*α radiation
μ = 0.23 mm^−1^
*T* = 90 (2) K
0.40 × 0.35 × 0.35 mm

#### Data collection

Bruker SMART APEX CCD area-detector diffractometer
Absorption correction: multi-scan (*SADABS*; Sheldrick, 1996 ▶) *T*_min_ = 0.874, *T*_max_ = 0.923
23047 measured reflections
4553 independent reflections
4540 reflections with *I* > 2σ(*I*)
*R*_int_ = 0.019

#### Refinement

*R*[*F* ^2^ > 2σ(*F* ^2^)] = 0.032
*wR*(*F* ^2^) = 0.087
*S* = 0.85
4553 reflections
237 parameters
H-atom parameters constrained
Δρ_max_ = 0.46 e Å^−3^
Δρ_min_ = −0.21 e Å^−3^
Absolute structure: Flack (1983 ▶), 1920 Friedel pairs
Flack parameter: 0.03 (4)

### 

Data collection: *SMART* (Bruker, 1998 ▶); cell refinement: *SAINT-Plus* (Bruker, 1998 ▶);; data reduction: *SAINT-Plus*; program(s) used to solve structure: *SHELXS97* (Sheldrick, 2008 ▶); program(s) used to refine structure: *SHELXL97* (Sheldrick, 2008 ▶); molecular graphics: *PLATON* (Spek, 2003 ▶); software used to prepare material for publication: *SHELXL97*.

## Supplementary Material

Crystal structure: contains datablocks I, global. DOI: 10.1107/S160053680800528X/pv2069sup1.cif

Structure factors: contains datablocks I. DOI: 10.1107/S160053680800528X/pv2069Isup2.hkl

Additional supplementary materials:  crystallographic information; 3D view; checkCIF report

## Figures and Tables

**Table 1:** Hydrogen-bond geometry (Å, °)

*D*—H⋯*A*	*D*—H	H⋯*A*	*D*⋯*A*	*D*—H⋯*A*
N10—H10*A*⋯O1	0.91	1.96	2.845 (2)	166
N10—H10*C*⋯Cl	0.91	2.31	3.112 (1)	147
N10—H10*B*⋯O18^i^	0.91	1.94	2.755 (1)	148
O1—H2⋯Cl^ii^	0.77	2.43	3.201 (1)	177
N30—H30⋯Cl^iii^	0.88	2.43	3.299 (1)	171
O1—H1⋯Cl^iv^	0.82	2.33	3.139 (1)	165

Symmetry codes: (i) 
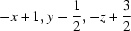
; (ii) 
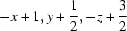
; (iii) 
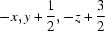
; (iv) 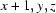.

**Fig. 1.**
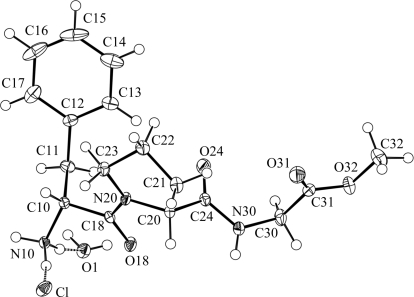
A view of (I) with displacement ellipsoids drawn at the 50% probability level with the aid of PLATON (Spek, 2003). Dotted lines represent hydrogen bonds.

**Fig. 2.**
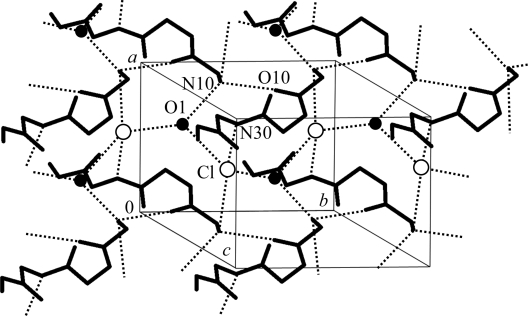
Packing diagram of (I). Side chains of amino acids are omitted for clarity. Dotted lines represent hydrogen bonds. Circles and filled-circles represent chloride ion (Cl) and water (O1) molecules.

## supplementary crystallographic information

### Comment

The β-turn structures were formed at D-Pro residue in a Gramicidin S and its analogue (Doi *et al.*, 2001; Yamada *et al.*, 2002; Llamas-Saiz, *et al.*, 2007), and a motif including D-Pro promoted β-hairpin in the protein GB1 analogue (Espinosa & Gellman, 2000). A tripeptide motif of Boc–Phe–D-Pro–Gly–OMe (Boc = t-butyloxycarbonyl; OMe = methylester) was designed from these peptides, and the β-turn structure was elucidated (Doi *et al.*, 2007). Moreover, the CD spectra of Gramicidin S analogues suggested that the chiral combination of Phe and Pro residues contributes to the β-turn formation (Tamaki *et al.*, 1985). Title peptide (I) was designed to highlight the chirality of the Phe residue in this tripeptide β-turn motif.

The molecular structure of (I) is shown in Fig. 1. The peptide is a chloride salt and its N-terminal (N10 atom) is protonated. The peptide molecule is somewhat extended, but the structure is not classified to any typical secondary structures from torsion angles. The Pro residue shows a ring puckering with amplitude of Q2 = 0.361 (2) Å and phase of φ2 = 293.1 (2) ° (Cremer & Pople, 1975), which is slightly different from those of the β-turn structure of Boc–Phe–D-Pro–Gly–OMe (Doi *et al.*, 2007).

A peptide-peptide hydrogen bond is formed between N10 and O18 atoms. This interaction makes the molecular arrangement propagated along the *b* axis, but no sheet structure is created (Fig. 2). Molecular packing is stabilized by the interactions with chloride ion (Cl) and water molecule (O1).

CD spectra of (I) showed no clear proof of special structures existed in acetonitril solution (data not shown), and the structure of (I) was somewhat extended. In contrast to the β-turn structure of the diastereomeric tripeptide (Boc–Phe–D-Pro–Gly–OMe), these results indicate that the chirality of Phe different from that of Pro is important for folding of this tripeptide motif.

### Experimental

The title compound was synthesized by a conventional liquid-phase method and the protected peptide, Boc–D-Phe–D-Pro–Gly–OMe (Boc = t-Butyloxycarboxy; OMe = methylester), was obtained. Boc group was removed by using HCl/dioxane, and the hydrocloride salt was obtained. Crystals were grown from aqueous acetonitrile solutions by vapor diffusion method.

### Refinement

The non-H atoms were refined anisotropically. H atoms were treated as riding atoms with distances C—H = 0.95–1.00 Å, N—H (–NH_3_^+^) = 0.91 Å and N—H (CONH) = 0.88 Å; *U*_iso_(H) = 1.2*U*_iso_(C), *U*_iso_(H) = 1.5*U*_eq_(C_methyl_), *U*_iso_(H) = 1.2*U*_eq_(N_CONH_) and *U*_iso_(H) = 1.5*U*_eq_(N_NH3_). H atoms of the water molecule were found in a difference Fourier map considering hydrogen-bond networks and fixed during refinements with *U*_iso_(H) = 1.2*U*_eq_(O). The absolute structure was based on the starting materials and was established by Flack parameter.

### Crystal data

**Table:**

C_17_H_24_N_3_O_4_^+^·Cl^–^·H_2_O	*F*_000_ = 824
*M**_r_* = 387.86	*D*_x_ = 1.334 Mg m^−^^3^
Orthorhombic, *P*2_1_2_1_2_1_	Mo *K*α radiation λ = 0.71073 Å
Hall symbol: P 2ac 2ab	Cell parameters from 8392 reflections
*a* = 7.3707 (5) Å	θ = 2.3–28.3º
*b* = 9.6667 (7) Å	µ = 0.23 mm^−^^1^
*c* = 27.099 (2) Å	*T* = 90 (2) K
*V* = 1930.8 (2) Å^3^	Cubic, colourless
*Z* = 4	0.40 × 0.35 × 0.35 mm

### Data collection

**Table:**

Bruker SMART APEX CCD area-detector diffractometer	4553 independent reflections
Radiation source: MacScience, M18XCE rotating anode	4540 reflections with *I* > 2σ(*I*)
Monochromator: graphite	*R*_int_ = 0.020
Detector resolution: 8.366 pixels mm^-1^	θ_max_ = 27.9º
*T* = 90(2) K	θ_min_ = 2.6º
ω–scan	*h* = −9→9
Absorption correction: multi-scan(SADABS; Sheldrick, 1996)	*k* = −12→12
*T*_min_ = 0.874, *T*_max_ = 0.923	*l* = −35→35
23047 measured reflections	

### Refinement

**Table:**

Refinement on *F*^2^	Hydrogen site location: inferred from neighbouring sites
Least-squares matrix: full	H-atom parameters constrained
*R*[*F*^2^ > 2σ(*F*^2^)] = 0.032	*w* = 1/[σ^2^(*F*_o_^2^) + (0.0703*P*)^2^ + 0.8632*P*] where *P* = (*F*_o_^2^ + 2*F*_c_^2^)/3
*wR*(*F*^2^) = 0.088	(Δ/σ)_max_ = 0.001
*S* = 0.85	Δρ_max_ = 0.46 e Å^−^^3^
4553 reflections	Δρ_min_ = −0.21 e Å^−^^3^
237 parameters	Extinction correction: none
Primary atom site location: structure-invariant direct methods	Absolute structure: Flack (1983), 1920 Friedel pairs
Secondary atom site location: difference Fourier map	Flack parameter: 0.03 (4)

### Special details

**Table:**

Geometry. Cremer & Pople Puckering Parameters [D. Cremer & J.A. Pople, J.Amer.Chem.Soc., 97, (1975), 1354–1358] ——————————————————————- Q(2) = 0.3608 (15) A ng., Phi(2) = 293.1 (2) DegThe equation of the plane is of the form: P * *x* + Q * *y* + *R* * *z* - S = 0 where P, Q, *R*, S are constants and *x*, *y*, *z* are fractional coordinates.P = 5.153 (2), Q = 1.935 (5), *R* = -18.602 (8), S = -9.378 (8) Atom Distance *x y z X* Y *Z* * O(18): -0.0397 (9) 0.4138 0.9441 0.7191 3.0500 9.1267 19.4869 * N(20): 0.0350 (11) 0.2674 0.8185 0.6615 1.9711 7.9123 17.9252 * C(10): 0.0337 (12) 0.4982 0.7074 0.7139 3.6722 6.8378 19.3462 * C(18): -0.0103 (12) 0.3858 0.8324 0.6981 2.8434 8.0464 18.9186 * C(20): 0.0268 (13) 0.1632 0.9399 0.6457 1.2030 9.0862 17.4973 * C(23): -0.0454 (14) 0.2080 0.6914 0.6361 1.5330 6.6837 17.2377P = 4.443 (3), Q = 0.711 (4), *R* = 21.531 (8), S = 15.452 (5) Atom Distance *x y z X* Y *Z* * O(24): 0.1064 (11) 0.4222 1.0289 0.6015 3.1119 9.9466 16.2998 * N(30): 0.0315 (11) 0.2213 1.1812 0.6344 1.6313 11.4182 17.1924 * C(20): -0.1560 (13) 0.1632 0.9399 0.6457 1.2030 9.0862 17.4973 * C(24): 0.0232 (13) 0.2849 1.0527 0.6252 2.1002 10.1765 16.9412 * C(30): -0.1661 (14) 0.2878 1.2994 0.6076 2.1213 12.5607 16.4662 * H(30): 0.16108 0.1378 1.1928 0.6573 1.0157 11.5304 17.8122
Refinement. Refinement of *F*^2^ against ALL reflections. The weighted *R*-factor *wR* and goodness of fit *S* are based on *F*^2^, conventional *R*-factors *R* are based on *F*, with *F* set to zero for negative *F*^2^. The threshold expression of *F*^2^ > σ(*F*^2^) is used only for calculating *R*-factors(gt) *etc*. and is not relevant to the choice of reflections for refinement. *R*-factors based on *F*^2^ are statistically about twice as large as those based on *F*, and *R*- factors based on ALL data will be even larger.

### Fractional atomic coordinates and isotropic or equivalent isotropic displacement parameters (Å^2^)

**Table:**

	*x*	*y*	*z*	*U*_iso_*/*U*_eq_	
Cl	0.11272 (5)	0.69212 (3)	0.784634 (14)	0.02274 (9)	
N10	0.52851 (16)	0.72346 (11)	0.76814 (4)	0.0135 (2)	
H10A	0.6034	0.7967	0.7737	0.020*	
H10B	0.5802	0.6451	0.7803	0.020*	
H10C	0.4203	0.7384	0.7834	0.020*	
C10	0.49822 (17)	0.70736 (13)	0.71391 (4)	0.0131 (2)	
H10	0.4307	0.6198	0.7070	0.016*	
C11	0.68581 (19)	0.70458 (16)	0.68855 (5)	0.0175 (3)	
H11A	0.7301	0.8008	0.6851	0.021*	
H11B	0.7720	0.6545	0.7101	0.021*	
C12	0.68628 (19)	0.63705 (14)	0.63820 (5)	0.0165 (3)	
C13	0.6299 (2)	0.70899 (16)	0.59619 (5)	0.0199 (3)	
H13	0.5899	0.8021	0.5990	0.024*	
C14	0.6322 (2)	0.6449 (2)	0.55034 (6)	0.0310 (4)	
H14	0.5924	0.6939	0.5219	0.037*	
C15	0.6927 (3)	0.5092 (2)	0.54597 (7)	0.0399 (5)	
H15	0.6951	0.4658	0.5145	0.048*	
C16	0.7488 (3)	0.43792 (18)	0.58701 (8)	0.0391 (5)	
H16	0.7901	0.3452	0.5839	0.047*	
C17	0.7457 (2)	0.50079 (16)	0.63329 (7)	0.0261 (3)	
H17	0.7841	0.4507	0.6616	0.031*	
C18	0.38577 (18)	0.83238 (13)	0.69813 (4)	0.0132 (2)	
O18	0.41380 (14)	0.94414 (10)	0.71910 (3)	0.0177 (2)	
N20	0.26742 (16)	0.81851 (11)	0.66147 (4)	0.0138 (2)	
C20	0.16322 (18)	0.93995 (13)	0.64568 (5)	0.0140 (3)	
H20	0.0887	0.9764	0.6736	0.017*	
C22	0.1285 (2)	0.74806 (14)	0.58840 (5)	0.0187 (3)	
H22A	0.0379	0.6835	0.5744	0.022*	
H22B	0.2248	0.7646	0.5636	0.022*	
C21	0.0392 (2)	0.88425 (14)	0.60427 (5)	0.0183 (3)	
H21A	−0.0852	0.8679	0.6167	0.022*	
H21B	0.0335	0.9501	0.5763	0.022*	
C23	0.20798 (19)	0.69142 (14)	0.63610 (5)	0.0160 (3)	
H23A	0.1153	0.6413	0.6556	0.019*	
H23B	0.3115	0.6289	0.6295	0.019*	
C24	0.28494 (18)	1.05274 (13)	0.62516 (5)	0.0143 (2)	
O24	0.42220 (15)	1.02895 (11)	0.60149 (4)	0.0226 (2)	
N30	0.22132 (17)	1.18119 (12)	0.63443 (4)	0.0175 (2)	
H30	0.1378	1.1928	0.6573	0.021*	
C30	0.2878 (2)	1.29938 (15)	0.60763 (5)	0.0206 (3)	
H30A	0.4206	1.2903	0.6031	0.025*	
H30B	0.2650	1.3841	0.6272	0.025*	
C31	0.1982 (2)	1.31382 (14)	0.55761 (5)	0.0183 (3)	
O31	0.09911 (18)	1.22970 (12)	0.53927 (4)	0.0269 (2)	
O32	0.24639 (17)	1.43362 (11)	0.53707 (4)	0.0248 (2)	
C32	0.1767 (3)	1.4570 (2)	0.48750 (6)	0.0367 (4)	
H32A	0.2270	1.3875	0.4650	0.055*	
H32B	0.2121	1.5496	0.4763	0.055*	
H32C	0.0441	1.4498	0.4878	0.055*	
O1	0.81122 (14)	0.92109 (10)	0.78001 (4)	0.0195 (2)	
H1	0.9029	0.8722	0.7787	0.023*	
H2	0.8324	0.9851	0.7638	0.023*	

### Atomic displacement parameters (Å^2^)

**Table:**

	*U*^11^	*U*^22^	*U*^33^	*U*^12^	*U*^13^	*U*^23^
Cl	0.01806 (16)	0.01737 (15)	0.03278 (18)	−0.00004 (13)	0.00761 (14)	−0.00212 (13)
N10	0.0160 (5)	0.0120 (5)	0.0123 (5)	0.0004 (4)	−0.0018 (4)	0.0010 (4)
C10	0.0151 (5)	0.0122 (5)	0.0119 (5)	0.0002 (5)	−0.0010 (5)	0.0010 (5)
C11	0.0148 (6)	0.0220 (7)	0.0157 (6)	−0.0005 (5)	0.0005 (5)	−0.0006 (5)
C12	0.0135 (5)	0.0168 (6)	0.0191 (6)	−0.0028 (5)	0.0032 (5)	−0.0043 (5)
C13	0.0174 (6)	0.0246 (7)	0.0178 (6)	−0.0043 (6)	0.0012 (5)	−0.0029 (5)
C14	0.0218 (7)	0.0511 (10)	0.0202 (7)	−0.0096 (8)	0.0021 (6)	−0.0081 (7)
C15	0.0281 (8)	0.0539 (12)	0.0375 (9)	−0.0133 (9)	0.0105 (7)	−0.0320 (9)
C16	0.0241 (8)	0.0245 (8)	0.0686 (13)	−0.0067 (7)	0.0142 (9)	−0.0251 (9)
C17	0.0175 (6)	0.0179 (7)	0.0429 (9)	−0.0013 (6)	0.0049 (7)	−0.0024 (6)
C18	0.0145 (5)	0.0123 (5)	0.0127 (5)	−0.0018 (5)	0.0024 (5)	0.0026 (4)
O18	0.0226 (5)	0.0122 (4)	0.0182 (4)	−0.0010 (4)	−0.0045 (4)	0.0000 (4)
N20	0.0166 (5)	0.0092 (5)	0.0157 (5)	−0.0006 (4)	−0.0014 (4)	0.0010 (4)
C20	0.0162 (6)	0.0119 (6)	0.0140 (5)	0.0011 (5)	0.0000 (5)	0.0023 (4)
C22	0.0217 (7)	0.0187 (6)	0.0157 (6)	−0.0025 (5)	−0.0052 (5)	−0.0013 (5)
C21	0.0182 (6)	0.0174 (6)	0.0194 (6)	−0.0035 (5)	−0.0053 (5)	0.0025 (5)
C23	0.0174 (6)	0.0124 (6)	0.0183 (6)	−0.0031 (5)	−0.0027 (5)	−0.0003 (5)
C24	0.0170 (6)	0.0130 (6)	0.0130 (5)	−0.0010 (5)	−0.0026 (5)	0.0017 (5)
O24	0.0220 (5)	0.0192 (5)	0.0266 (5)	−0.0012 (4)	0.0077 (4)	0.0041 (4)
N30	0.0227 (6)	0.0128 (5)	0.0169 (5)	−0.0005 (5)	0.0011 (4)	0.0021 (4)
C30	0.0268 (7)	0.0137 (6)	0.0214 (6)	−0.0047 (6)	−0.0040 (5)	0.0038 (5)
C31	0.0215 (6)	0.0153 (6)	0.0181 (6)	0.0031 (6)	0.0039 (5)	0.0006 (5)
O31	0.0353 (6)	0.0221 (5)	0.0233 (5)	−0.0015 (5)	−0.0060 (5)	−0.0025 (4)
O32	0.0299 (6)	0.0211 (5)	0.0233 (5)	0.0000 (5)	−0.0007 (5)	0.0089 (4)
C32	0.0483 (11)	0.0399 (9)	0.0218 (7)	0.0074 (9)	0.0005 (7)	0.0101 (7)
O1	0.0186 (5)	0.0172 (4)	0.0227 (5)	0.0001 (4)	0.0005 (4)	0.0003 (4)

### Geometric parameters (Å, °)

**Table:**

N10—C10	1.4947 (16)	C20—C21	1.5441 (18)
N10—H10A	0.9100	C20—H20	1.0000
N10—H10B	0.9100	C22—C23	1.5209 (18)
N10—H10C	0.9100	C22—C21	1.533 (2)
C10—C18	1.5265 (17)	C22—H22A	0.9900
C10—C11	1.5442 (18)	C22—H22B	0.9900
C10—H10	1.0000	C21—H21A	0.9900
C11—C12	1.5125 (18)	C21—H21B	0.9900
C11—H11A	0.9900	C23—H23A	0.9900
C11—H11B	0.9900	C23—H23B	0.9900
C12—C17	1.394 (2)	C24—O24	1.2197 (17)
C12—C13	1.397 (2)	C24—N30	1.3509 (17)
C13—C14	1.388 (2)	N30—C30	1.4397 (17)
C13—H13	0.9500	N30—H30	0.8800
C14—C15	1.391 (3)	C30—C31	1.5144 (19)
C14—H14	0.9500	C30—H30A	0.9900
C15—C16	1.372 (3)	C30—H30B	0.9900
C15—H15	0.9500	C31—O31	1.2006 (19)
C16—C17	1.394 (3)	C31—O32	1.3329 (17)
C16—H16	0.9500	O32—C32	1.456 (2)
C17—H17	0.9500	C32—H32A	0.9800
C18—O18	1.2381 (16)	C32—H32B	0.9800
C18—N20	1.3289 (17)	C32—H32C	0.9800
N20—C20	1.4666 (16)	O1—H1	0.825
N20—C23	1.4745 (17)	O1—H2	0.775
C20—C24	1.5176 (18)		
			
C10—N10—H10A	109.5	N20—C20—H20	110.4
C10—N10—H10B	109.5	C24—C20—H20	110.4
H10A—N10—H10B	109.5	C21—C20—H20	110.4
C10—N10—H10C	109.5	C23—C22—C21	103.65 (11)
H10A—N10—H10C	109.5	C23—C22—H22A	111.0
H10B—N10—H10C	109.5	C21—C22—H22A	111.0
N10—C10—C18	105.90 (10)	C23—C22—H22B	111.0
N10—C10—C11	107.80 (10)	C21—C22—H22B	111.0
C18—C10—C11	112.04 (10)	H22A—C22—H22B	109.0
N10—C10—H10	110.3	C22—C21—C20	104.43 (11)
C18—C10—H10	110.3	C22—C21—H21A	110.9
C11—C10—H10	110.3	C20—C21—H21A	110.9
C12—C11—C10	114.26 (11)	C22—C21—H21B	110.9
C12—C11—H11A	108.7	C20—C21—H21B	110.9
C10—C11—H11A	108.7	H21A—C21—H21B	108.9
C12—C11—H11B	108.7	N20—C23—C22	102.16 (10)
C10—C11—H11B	108.7	N20—C23—H23A	111.3
H11A—C11—H11B	107.6	C22—C23—H23A	111.3
C17—C12—C13	119.06 (14)	N20—C23—H23B	111.3
C17—C12—C11	119.64 (14)	C22—C23—H23B	111.3
C13—C12—C11	121.30 (13)	H23A—C23—H23B	109.2
C14—C13—C12	120.25 (15)	O24—C24—N30	123.99 (13)
C14—C13—H13	119.9	O24—C24—C20	123.20 (12)
C12—C13—H13	119.9	N30—C24—C20	112.77 (12)
C13—C14—C15	120.05 (18)	C24—N30—C30	121.16 (12)
C13—C14—H14	120.0	C24—N30—H30	119.4
C15—C14—H14	120.0	C30—N30—H30	119.4
C16—C15—C14	120.10 (16)	N30—C30—C31	112.10 (12)
C16—C15—H15	120.0	N30—C30—H30A	109.2
C14—C15—H15	120.0	C31—C30—H30A	109.2
C15—C16—C17	120.34 (17)	N30—C30—H30B	109.2
C15—C16—H16	119.8	C31—C30—H30B	109.2
C17—C16—H16	119.8	H30A—C30—H30B	107.9
C16—C17—C12	120.20 (17)	O31—C31—O32	125.29 (13)
C16—C17—H17	119.9	O31—C31—C30	124.98 (13)
C12—C17—H17	119.9	O32—C31—C30	109.73 (12)
O18—C18—N20	122.73 (12)	C31—O32—C32	115.22 (13)
O18—C18—C10	118.15 (11)	O32—C32—H32A	109.5
N20—C18—C10	119.07 (11)	O32—C32—H32B	109.5
C18—N20—C20	118.75 (11)	H32A—C32—H32B	109.5
C18—N20—C23	128.88 (11)	O32—C32—H32C	109.5
C20—N20—C23	112.05 (10)	H32A—C32—H32C	109.5
N20—C20—C24	111.86 (11)	H32B—C32—H32C	109.5
N20—C20—C21	104.05 (10)	H1—O1—H2	105.56
C24—C20—C21	109.51 (11)		
			
N10—C10—C11—C12	158.2 (1)	C23—N20—C20—C24	−122.61 (12)
C18—C10—C11—C12	−85.66 (14)	C18—N20—C20—C21	−178.58 (11)
C10—C11—C12—C17	−99.94 (15)	C23—N20—C20—C21	−4.49 (14)
C10—C11—C12—C13	80.71 (17)	C23—C22—C21—C20	34.16 (14)
C17—C12—C13—C14	0.4 (2)	N20—C20—C21—C22	−18.6 (1)
C11—C12—C13—C14	179.76 (13)	C24—C20—C21—C22	101.18 (12)
C12—C13—C14—C15	−0.8 (2)	C18—N20—C23—C22	−160.99 (13)
C13—C14—C15—C16	0.5 (3)	C20—N20—C23—C22	25.66 (14)
C14—C15—C16—C17	0.0 (3)	C21—C22—C23—N20	−36.02 (13)
C15—C16—C17—C12	−0.4 (3)	N20—C20—C24—O24	35.04 (17)
C13—C12—C17—C16	0.1 (2)	C21—C20—C24—O24	−79.78 (16)
C11—C12—C17—C16	−179.22 (14)	N20—C20—C24—N30	−147.2 (1)
N10—C10—C18—O18	34.80 (15)	C21—C20—C24—N30	98.00 (13)
C11—C10—C18—O18	−82.47 (14)	O24—C24—N30—C30	14.4 (2)
N10—C10—C18—N20	−147.7 (1)	C20—C24—N30—C30	−163.4 (1)
C11—C10—C18—N20	95.02 (14)	C24—N30—C30—C31	80.9 (2)
O18—C18—N20—C20	−1.09 (19)	N30—C30—C31—O31	−8.2 (2)
C10—C18—N20—C20	−178.5 (1)	N30—C30—C31—O32	172.0 (1)
O18—C18—N20—C23	−174.05 (12)	O31—C31—O32—C32	−3.0 (2)
C10—C18—N20—C23	8.58 (19)	C30—C31—O32—C32	176.89 (14)
C18—N20—C20—C24	63.3 (2)		

### Hydrogen-bond geometry (Å, °)

**Table:**

*D*—H···*A*	*D*—H	H···*A*	*D*···*A*	*D*—H···*A*
N10—H10A···O1	0.91	1.96	2.845 (2)	166
N10—H10C···Cl	0.91	2.31	3.112 (1)	147
N10—H10B···O18^i^	0.91	1.94	2.755 (1)	148
O1—H2···Cl^ii^	0.77	2.43	3.201 (1)	177
N30—H30···Cl^iii^	0.88	2.43	3.299 (1)	171
O1—H1···Cl^iv^	0.82	2.33	3.139 (1)	165

Symmetry codes: (i) −*x*+1, *y*−1/2, −*z*+3/2; (ii) −*x*+1, *y*+1/2, −*z*+3/2; (iii) −*x*, *y*+1/2, −*z*+3/2; (iv) *x*+1, *y*, *z*.
